# Upcoming therapies for steatotic liver disease: Translating breakthroughs into clinical practice

**DOI:** 10.1111/joim.70143

**Published:** 2026-08-31

**Authors:** Philip N. Newsome, Saima Ajaz

**Affiliations:** ^1^ Institute of Liver Studies Kings College Hospital London UK; ^2^ King's College London London UK

**Keywords:** emerging therapies, incretin therapy, liver fibrosis, MASH, MASLD, noninvasive tests

## Abstract

Metabolic dysfunction‐associated steatotic liver disease (MASLD) is the most common chronic liver disease worldwide and a leading cause of cirrhosis, hepatocellular carcinoma, and cardiometabolic morbidity. Often clinically silent until advanced fibrosis develops, management has largely relied on lifestyle interventions, with limited pharmacological options and inconsistent risk stratification. Recent advances in noninvasive diagnostics and targeted therapies are beginning to transform this approach. This review examines the evolving management paradigm of MASLD and metabolic dysfunction‐associated steatohepatitis (MASH), focusing on practical implementation of fibrosis‐based risk stratification, structured care pathways, and newly approved or late‐stage pharmacological therapies. Recent clinical trials demonstrate that therapies targeting metabolic dysfunction and hepatic fibrogenesis can improve steatohepatitis and fibrosis, particularly in patients with advanced disease. Incretin‐based therapies have shown MASH resolution in approximately 40%–60% of patients, accompanied by weight loss of 10%–20%, whereas thyroid hormone receptor‐β agonists have demonstrated substantial reductions in liver fat and improvements in fibrosis‐related endpoints. Emerging fibroblast growth factor analogs and combination therapeutic approaches suggest additive benefit through complementary metabolic and anti‐fibrotic mechanisms. Together, these advances support treatment strategies guided by fibrosis risk rather than steatosis alone. MASLD care is therefore moving from reactive disease management toward structured pathways that integrate scalable diagnostics with disease‐modifying therapy, offering the potential to alter disease progression and reduce the burden of advanced liver disease.

## Graphical abstract

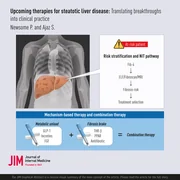

## Introduction

In 2023, international liver societies agreed to rename nonalcoholic fatty liver disease (NAFLD) as metabolic dysfunction‐associated steatotic liver disease (MASLD), with the term metabolic dysfunction‐associated steatohepatitis (MASH) replacing NASH for the inflammatory, fibrotic subtype. This updated nomenclature emphasizes that fatty liver disease is fundamentally driven by metabolic dysfunction rather than defined by the absence of alcohol [1, 2]. This nomenclature shift comes amid an alarming global rise in MASLD/MASH. Steatotic liver disease (SLD) is now the most common chronic liver condition worldwide, affecting roughly one‐third of adults. Its prevalence has climbed in step with the obesity and Type 2 diabetes epidemics, reaching as high as 30%–40% of the population in some regions [1, 3].

A major challenge in addressing this burden is that MASLD remains largely clinically silent until advanced fibrosis develops, resulting in many individuals remaining undiagnosed until late stages of disease. This creates a fundamental tension for healthcare systems, which must identify those at risk of progression early while avoiding overdiagnosis in a highly prevalent condition [4, 5].

By 2040, MASLD prevalence is projected to surpass 55% globally, with the burden greatest in younger populations and in rapidly urbanizing regions such as Asia and the Middle East [1, 3]. A considerable subset of individuals with MASLD will progress to the more aggressive stage of MASH over time, especially when metabolic risk factors remain uncontrolled. As a result, MASLD has become a major contributor to serious liver outcomes globally. Rates of advanced fibrosis, cirrhosis, and hepatocellular carcinoma (HCC) due to MASLD/MASH are steadily rising in parallel with the global surge in obesity and metabolic disease [1, 6]. A key implication for treatment is that fibrosis stage, rather than steatosis alone, is the principal determinant of liver‐related risk and therefore the primary target for disease‐modifying therapy.

The epidemiology of end‐stage liver disease and hepatocellular carcinoma (HCC) has shifted accordingly. With viral hepatitis increasingly controlled, MASLD/MASH has emerged as a leading cause of cirrhosis and liver cancer. In the United States, MASH now rivals alcoholic liver disease as the top indication for liver transplantation and has surpassed hepatitis C as the leading cause of HCC among transplant candidates [1, 6]. Similar patterns are seen globally as metabolic liver disease replaces viral hepatitis as a dominant driver of liver failure. These trends underscore that MASLD is not a benign condition, and if left unchecked, it can progress to life‐threatening liver disease and cancer, creating a public health challenge. For therapeutics, this shift also raises the bar from short‐term improvements in steatosis or enzymes toward anti‐fibrotic efficacy and, ultimately, evidence of reduced clinical events.

Moreover, MASLD carries risks beyond the liver due to its tight links with cardiometabolic disease. Most patients with MASLD have one or more features of metabolic syndrome, and consequently, they face elevated rates of cardiovascular morbidity and mortality. Indeed, cardiovascular disease is the leading cause of death in this patient population, exceeding liver‐related deaths [1, 2]. This reality means that effective management of MASLD requires comprehensive care. Alongside monitoring liver fibrosis and function, clinicians must aggressively treat associated metabolic conditions such as promoting weight loss, improving glycemic control, and managing lipids and blood pressure to reduce overall morbidity [2]. In practical terms, the most effective treatment strategies will be those that improve both liver histology and cardiometabolic risk, which partly explains the momentum behind incretin‐based and FGF21‐based agents.

Despite the enormous burden of MASLD/MASH, no medications were approved for these conditions until very recently. For decades, lifestyle modification remained the cornerstone of therapy, with weight loss via diet and exercise often producing improvements in liver fat and inflammation. However, sustained weight loss is difficult to achieve for many, and a large proportion of patients have progressed to advanced fibrosis despite optimal lifestyle efforts [3, 6]. This lack of specific treatment options has long highlighted an urgent unmet need for pharmacological interventions to halt or reverse disease progression. Even with effective drugs, non‐pharmacological therapies remain disease‐modifying and will continue to sit alongside pharmacotherapy rather than be replaced by it.

That landscape began to change in March 2024 with a breakthrough: Resmetirom, a selective thyroid hormone receptor‐β agonist, became the first drug approved by the US Food and Drug Administration (FDA) and subsequently in the European Union for MASH with fibrosis [3, 7, 8]. In clinical trials, resmetirom demonstrated the ability to resolve MASH and improve fibrosis, offering proof that targeted therapy can modify the course of this disease [3, 7]. Its approval marks the start of a new era in which pharmacotherapy can be paired with lifestyle measures to more effectively treat MASLD. This momentum continued with the approval of semaglutide (Wegovy) by the US FDA on August 15, 2025 for the treatment of MASH in adults with moderate‐to‐advanced fibrosis, representing the second disease‐specific pharmacotherapy in this space [9], followed by its approval in the European Union in March–April 2026 [10]. Furthermore, several other promising agents are in advanced clinical trials and may soon expand the therapeutic landscape. These include metabolic treatments like Glucagon‐like peptide‐1 (GLP‐1) receptor agonists, which have shown significant reductions in liver fat and necroinflammation alongside weight loss, as well as drugs targeting fibrogenesis and inflammation (such as pan‐peroxisome proliferator‐activated receptor [PPAR] agonists and FGF21 analogs) [2, 3, 6]. It is anticipated that multiple mechanisms of action addressing the metabolic, inflammatory, and fibrotic aspects of MASH will eventually be combined for optimal outcomes [3, 6]. This emerging portfolio can be viewed as two complementary treatment intents: metabolic unloading to remove the upstream driver and a fibrosis brake to accelerate regression in higher risk disease.

This review will examine the major therapeutic breakthroughs in the management of MASLD/MASH and discuss how these advances can be translated into clinical practice. We will summarize the key efficacy and safety data from recent clinical trials of new agents (including resmetirom and other late‐stage candidates) and provide practical insights on patient selection, monitoring, and integrating these therapies with lifestyle interventions. Our aim is to provide an up‐to‐date understanding of the evolving treatment landscape and to outline a roadmap for incorporating these breakthroughs into patient care.

## Management of MASLD in primary care and community hepatology

The growing burden of MASLD means that general practitioners (GPs) are increasingly encountering patients with incidental steatotic liver on imaging or mild liver enzyme elevations. Early identification and triage of at‐risk patients is essential because timely intervention can prevent progression to cirrhosis and its complications [11, 12, 13]. Importantly, the stage of fibrosis (not the mere presence of steatosis) is the key prognostic factor for liver outcomes in MASLD. Patients with advanced fibrosis (bridging fibrosis or cirrhosis) have dramatically higher risks of liver‐related morbidity and mortality, whereas those without significant fibrosis rarely develop end‐stage liver disease. Detecting advanced fibrosis early, before cirrhosis and decompensation occur, allows for referral and intervention at a stage when outcomes can be improved. Indeed, recent European guidelines emphasize that with new treatment options emerging, identifying at‐risk individuals with MASLD early is critical to positively influence the course of the disease and prevent clinical events. In summary, the sheer prevalence of MASLD and its silent but progressive nature make early case‐finding and risk stratification in primary care a high priority in order to triage patients appropriately and reduce the future burden of advanced liver disease. This risk stratification is now also treatment enabling; it identifies the subgroup most likely to benefit from disease‐modifying pharmacotherapy and from follow‐up strategies anchored to fibrosis risk rather than steatosis alone.

## Identifying high‐risk patients beyond lifestyle measures

Although lifestyle modification is the foundation of MASLD management for all patients, the challenge in primary care is to discern which patients may need more than lifestyle intervention. Not every patient with steatotic liver requires referral; the focus should be on those at risk of progressive disease or advanced fibrosis. Key risk factors that identify patients who warrant closer attention include the following sections.

### Evidence of significant fibrosis

Patients who already have significant fibrosis (stages F3–F4) on noninvasive assessment are at highest risk for liver‐related complications. These individuals require hepatology referral and multidisciplinary care (e.g., cirrhosis surveillance) rather than just lifestyle advice. Thus, any fibrosis risk scoring that suggests advanced fibrosis is a red flag [3, 13]. This group is also the priority population for liver‐directed therapies and for combination strategies where available evidence supports anti‐fibrotic benefit.

### Type 2 diabetes mellitus (T2DM)

Type 2 diabetes mellitus (T2DM) is the strongest metabolic predictor of MASH and fibrosis progression in MASLD. Approximately half of people with T2DM have MASLD, and they have a higher likelihood of advanced fibrosis and incident HCC than non‐diabetics with MASLD [1, 3]. Current guidelines consider all patients with T2DM and steatotic liver as high‐risk, meriting proactive fibrosis assessment. From a treatment perspective, T2DM also identifies patients most likely to benefit from incretin‐based therapy, with liver improvement alongside cardiometabolic risk reduction.

### Metabolic syndrome and multiple risk factors

Obesity (especially abdominal obesity) combined with one or more additional cardiometabolic risk factors (e.g., hypertension, dyslipidemia, and insulin resistance) confers a high‐risk of progressive liver disease. Importantly, risk appears to be synergistic rather than simply additive, with the coexistence of multiple metabolic abnormalities particularly Type 2 diabetes driving a disproportionate increase in advanced fibrosis risk, as demonstrated in a large contemporary analysis [14]. Consistent with this, a meta‐analysis [15] indicates that although obesity represents a key upstream driver, its effect size on liver‐related outcomes is modest in comparison to that of Type 2 diabetes, which is associated with more than a twofold increase in the risk of severe liver disease, thereby underscoring diabetes as a dominant determinant of disease progression.

The presence of metabolic syndrome features in a patient with MASLD should prompt fibrosis evaluation beyond simple lifestyle advice [1, 3]. This phenotype is the core target for metabolic unloading therapies that deliver sustained weight loss and improvements in insulin resistance. Collectively, these data support a risk‐stratified approach in which the intensity of assessment and intervention is guided not only by adiposity but also by the cumulative metabolic burden and presence of high‐impact drivers such as Type 2 diabetes.

### Age and demographic factors

Older age is a known risk factor for fibrosis progression. Men over 50 and postmenopausal women have higher likelihood of advanced fibrosis or cirrhosis from MASLD. Thus, an older patient with steatotic liver deserves careful fibrosis risk stratification. Family history of cirrhosis or HCC and certain ethnic backgrounds (e.g., South Asian) [16] may also heighten risk, though these are less emphasized in guidelines.

### Persistently abnormal liver enzymes

Although many patients with MASLD have normal or mildly elevated ALT/AST, the persistence of elevated liver enzymes in the context of metabolic risk factors is concerning. Elevated ALT/AST in MASLD correlates with greater likelihood of steatohepatitis and fibrosis [17]. Current EASL guidelines recommend that *any* patient with unexplained liver enzyme elevations plus metabolic risk (or imaging evidence of steatosis) should undergo fibrosis assessment [12]. Normal ALT, on the other hand, does not exclude advanced disease; hence, reliance on liver enzymes alone is insufficient. This is particularly important when selecting patients for treatment and monitoring response: Biochemical improvement is reassuring but cannot be assumed to track fibrosis regression.

### Other comorbidities

Patients with coexisting conditions, such as obstructive sleep apnea, polycystic ovary syndrome, or genetic predispositions (e.g., PNPLA3 variants), may have higher risk of MASH and fibrosis [18]. These factors are not standalone referral criteria, but they can heighten suspicion when present alongside the above risk factors.

In practice, primary care providers should have a low threshold to evaluate fibrosis risk in any patient with steatotic who has T2DM, metabolic syndrome, or other high‐risk features. These are the patients most likely to benefit from early intervention (beyond lifestyle) such as pharmacotherapy or specialist management. By contrast, a young patient with incidentally noted steatosis, no metabolic risk factors, and low fibrosis score can usually be managed with lifestyle measures in primary care. The overarching principle is to triage patients with MASLD by fibrosis risk because those with mild disease can be managed conservatively, whereas those with high fibrosis risk need escalation of care. This triage also provides a pragmatic bridge to therapy selection: metabolic agents for systemic risk reduction across the spectrum and liver‐directed agents, where fibrosis risk is established, and the benefit‐to‐risk profile is highest.

## Noninvasive testing algorithms in primary care

The advent of noninvasive tests (NITs) for liver fibrosis has revolutionized MASLD management in the community. Rather than referring all patients for invasive biopsy or specialist assessment, GPs can employ simple algorithms combining blood tests and imaging to stratify fibrosis risk. Recent European guidelines endorse a stepwise approach: start with an inexpensive blood‐based score in at‐risk individuals, then use more specific tests (elastography or patented biomarkers) only if needed. This approach efficiently rules out those without advanced fibrosis and pinpoints those likely to have advanced disease. The most widely used NITs in primary care include Fibrosis‐4 (FIB‐4), Enhanced Liver Fibrosis (ELF), vibration‐controlled transient elastography (VCTE), and emerging MRI‐based techniques.

FIB‐4 Index: This is a simple, non‐proprietary score calculated from four routine parameters (age, AST, ALT, and platelet count). It is recommended as the first‐line screening tool for fibrosis in MASLD because it is readily available and cost‐free. A low FIB‐4 result reliably excludes advanced fibrosis with high negative predictive value (NPV). For example, an FIB‐4 below 1.3 (using the standard cutoff for <65‐year‐olds) has an NPV around 90%–95% for advanced fibrosis [3, 13, 19]. In practical terms, if FIB‐4 is low, one can be reasonably confident the patient does not have F3–F4 fibrosis. Conversely, a high FIB‐4 suggests significant fibrosis at the traditional >2.67 cutoff, FIB‐4 has about 85% specificity for advanced fibrosis. Patients with FIB‐4 above this upper threshold are considered “rule‐in” for advanced disease and should be referred or further evaluated without delay. The limitation of FIB‐4 is the sizeable indeterminate “grey zone” (often 1.3–2.67) and reduced accuracy in older patients (age >65 tends to raise FIB‐4). Nonetheless, it remains a valuable triage test with one study showing FIB‐4 <1.3 could avoid unnecessary referrals in 50% of cases, whereas FIB‐4 >2.67 flagged roughly 25% as high‐risk [20]. Current practice is to use an age‐adjusted cutoff (e.g., <2.0 for seniors) and to proceed to a second‐line test for indeterminate results. Importantly, emerging evidence supports the use of serial or repeated FIB‐4 measurements rather than a single time‐point assessment. Longitudinal studies demonstrate that dynamic changes in FIB‐4 over time are independently associated with liver‐related outcomes, with rising trajectories identifying patients at increased risk of progression to advanced fibrosis and liver‐related events, even when baseline values are indeterminate [21, 22]. Conversely, stable or declining FIB‐4 values are associated with a lower risk profile, suggesting potential utility in monitoring disease evolution and response to intervention. This extends the role of FIB‐4 beyond a single assessment, enabling its use for both risk stratification and ongoing monitoring in MASLD. Beyond referral decisions, NITs increasingly act as treatment gates for eligibility and as longitudinal monitoring tools alongside clinical assessment.

### Enhanced liver fibrosis (ELF) test

ELF test is a serum‐based NIT that measures markers of extracellular matrix turnover consisting of hyaluronic acid, Procollagen III N‐terminal peptide (P3NP), and tissue inhibitor of metalloproteinases‐1 (TIMP‐1) to generate a composite fibrosis score. ELF is a validated second‐line NIT for fibrosis risk stratification in MASLD and is endorsed by NICE in the United Kingdom for use in clinical pathways [23]. An ELF score above established thresholds (typically 9.8–10.5) indicates a high probability of advanced fibrosis, whereas a low ELF score (<9) is associated with minimal fibrosis [24, 25]. ELF is commonly deployed after an initial screening test such as FIB‐4, particularly when transient elastography is unavailable or inconclusive. In terms of diagnostic performance, ELF is broadly comparable to VCTE for the detection of advanced fibrosis, with the advantage of requiring only a blood sample, although higher cost and limited availability may restrict its use in some primary care settings [25]. Current EASL guidelines recognize collagen biomarker panels such as ELF as acceptable alternatives to imaging‐based techniques for identifying advanced fibrosis and support their use in sequential testing algorithms [15].

Importantly, ELF is not specific to MASLD and reflects extracellular matrix turnover and fibrogenesis more broadly; therefore, elevated ELF values may also be observed in other chronic liver diseases, including viral hepatitis, alcohol‐related liver disease, autoimmune liver diseases, and cholestatic liver disorders [26, 27]. As a result, ELF should be interpreted within the overall clinical context, as elevated values do not necessarily distinguish MASLD‐related fibrosis from fibrosis due to other hepatic etiologies. Consequently, ELF is best utilized as part of a sequential noninvasive testing strategy rather than in isolation to improve diagnostic accuracy and reduce false‐positive classification of advanced fibrosis.

### Vibration‐controlled transient elastography (VCTE)

VCTE is an ultrasound‐based technique that measures liver stiffness (in kilopascals, kPa) as a surrogate for fibrosis and provides a controlled attenuation parameter for quantification of hepatic steatosis. It is widely used across Europe in specialist and community‐based settings and is commonly employed as a second‐line test following an abnormal FIB‐4. The principal advantages of VCTE are its speed (typically 5–10 min), point‐of‐care applicability, and validated accuracy for advanced fibrosis and cirrhosis across chronic liver diseases. In MASLD, liver stiffness values <8 kPa generally indicate absence of advanced fibrosis (F0–F2), whereas values >8–10 kPa suggest significant fibrosis (≥F3). According to the Baveno VI criteria, a liver stiffness measurement (LSM) ≥10 kPa is suggestive of compensated advanced chronic liver disease (cACLD), whereas ≥15 kPa is highly suggestive of cirrhosis and warrants cirrhosis‐specific management, including variceal screening [28, 29]. VCTE demonstrates excellent specificity for ruling in cirrhosis at higher cutoffs (approximately 95% at 15 kPa) and good NPV for excluding advanced fibrosis at lower thresholds, although intermediate values require clinical context. Transient elevations in liver stiffness may occur with hepatic inflammation, congestion, or recent food intake, and results should therefore be interpreted cautiously. Despite these limitations, VCTE remains a cornerstone of MASLD assessment and a key tool for guiding hepatology referral pathways [28, 29].

Emerging longitudinal data suggest that single VCTE measurements may overestimate fibrosis severity in some individuals because of biological and technical variability, with subsequent repeat measurements often regressing toward lower values (two new references). Population‐based studies, including the LiverScreen study, have shown that elevated VCTE values may decrease on repeat testing, particularly in intermediate ranges, highlighting the importance of sequential testing and repeat assessment before confirming advanced fibrosis or cirrhosis.

Beyond fibrosis staging, VCTE also appears to have prognostic utility. Recent longitudinal studies, including work by Mozes et al., have demonstrated that liver stiffness measured by VCTE predicts future major adverse liver ‐ outcomes (MALO), including hepatic decompensation, hepatocellular carcinoma, liver transplantation, and liver‐related mortality, with prognostic performance comparable to or in some settings exceeding liver biopsy [30]. This supports the evolving role of VCTE not only as a diagnostic tool, but also as a clinically meaningful biomarker for risk stratification and longitudinal disease monitoring in MASLD.

### MRI‐based techniques

Magnetic resonance offers highly accurate quantification of both liver fat and fibrosis. MRI‐PDFF (proton density fat fraction) can noninvasively measure hepatic steatosis, and MR elastography (MRE) can map tissue stiffness with even greater accuracy than VCTE. Additionally, emerging MRI‐based composite metrics (e.g., iron‐corrected T1 or LiverMultiScan) provide insights into liver fibro‐inflammation [31]. Although MRI‐based tools are increasingly recognized within MASLD care pathways, their role is currently mainly confined to specialist hepatology centers, tertiary referral pathways, and clinical trial settings rather than routine primary care screening, largely due to accessibility and infrastructure requirements. MRE has excellent performance (AUROC 0.90 for advanced fibrosis), but it requires referral to a tertiary center. Likewise, MRI‐PDFF can confirm steatotic liver and quantify fat reduction after interventions, but an ultrasound is usually sufficient for diagnosing steatosis in primary care. European guidelines acknowledge MRE as a valid noninvasive option alongside VCTE and indeed list MRE or MRI‐based scores as complementary tools for fibrosis staging and risk stratification. As MRI technology becomes more accessible, it may play a larger role in MASLD care pathways, but currently its main use in Europe is in specialized hepatology or clinical trial settings rather than frontline primary care screening.

### Comparative performance

It is important to recognize that no single NIT provides perfect diagnostic accuracy, and each approach is associated with inherent limitations in sensitivity, specificity, availability, and cost. Blood‐based scores (FIB‐4, NAFLD Fibrosis Score) are simple and sensitive but have lower specificity and can be confounded by age or comorbidities. Direct biomarkers like ELF improve specificity for fibrosis but cost more. VCTE gives a direct physical measure with good specificity but can overestimate fibrosis if done improperly or in certain contexts. MRI provides the highest accuracy but at high cost. Therefore, combining tests sequentially using a rule‐out test followed by a rule‐in test achieves the best balance. For example, one might use FIB‐4 (high sensitivity) to catch nearly all at‐risk patients, then VCTE or ELF (higher specificity) to confirm advanced fibrosis before referral. Such sequential algorithms have been shown to reduce unnecessary referrals while still capturing the vast majority of advanced fibrosis cases. Indeed, EASL's latest guidance strongly endorses clinical pathways built on sequential NIT application in primary care, as they are more cost‐effective and accurate than relying on liver enzymes. In summary, primary care clinicians now have a toolkit of NITs to triage MASLD: start broad with simple tests, then narrow in with specialized tests as this approach maximizes early detection of silent advanced disease while minimizing overburdening of specialists with low‐risk steatotic liver cases.

## Intelligent liver function testing and AI‐based triage

One innovative approach to improving MASLD management in primary care is the use of intelligent liver function testing (iLFT) and other AI‐driven triage tools [32]. These systems leverage algorithms to interpret routine lab results and risk factors, helping clinicians identify occult liver disease early and automate the next steps [33]. A flagship example is the iLFT pathway developed in NHS Tayside (Scotland). In the iLFT model, when a GP orders liver blood tests and any result is abnormal, the laboratory's computer system reflexively triggers a cascade of additional tests (e.g., hepatitis serologies, autoantibodies, ferritin, and ELF fibrosis panel) and computes an algorithmic diagnosis [34]. The GP then receives a consolidated report that indicates the likely diagnosis (MASLD, alcohol‐related, etc.) and whether advanced fibrosis is present, along with management recommendations. This approach is termed “intelligent” because it removes the need for GPs to determine which additional investigations are required, instead using an algorithmic framework that automatically evaluates a broad spectrum of liver disease etiologies and disease severity.

## Recommended triage and referral pathway

**Fig. 1:**
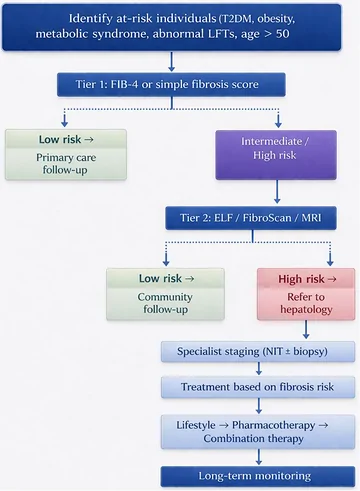
Proposed clinical pathway for the identification, risk stratification and management of MASLD/MASH. A two‐tier, non‐invasive assessment pathway for adults at increased risk of metabolic dysfunction‐associated steatotic liver disease (MASLD), including individuals with type 2 diabetes mellitus, obesity, metabolic syndrome, abnormal liver function tests, or age >50 years. Initial assessment using a simple fibrosis score (e.g. FIB‐4) identifies patients at low risk, who can be managed in primary care, and those at intermediate or high risk, who undergo second‐line assessment with enhanced liver fibrosis (ELF) testing, vibration‐controlled transient elastography, or magnetic resonance imaging (MRI). Patients identified as high risk are referred to hepatology for specialist staging using non‐invasive tests (with liver biopsy where indicated), followed by fibrosis‐stagedirected management, including lifestyle intervention, pharmacotherapy, or combination therapy, with ongoing long‐term monitoring.

Bringing the above elements together, current European best practice is to implement a standardized pathway for MASLD in primary care. A narrative summary of the recommended clinical pathway is as follows (Fig. 1 ):

1. Identify at‐risk individuals
2. Perform initial fibrosis risk assessment (Tier 1)
3. Perform additional noninvasive testing (Tier 2)
4. Refer to hepatology (specialist care)
5. Link fibrosis risk to treatment intensity, ranging from lifestyle and cardiometabolic optimization through to disease‐modifying pharmacotherapy and combination approaches in higher risk disease.

## Therapeutic landscape

**Fig. 2:**
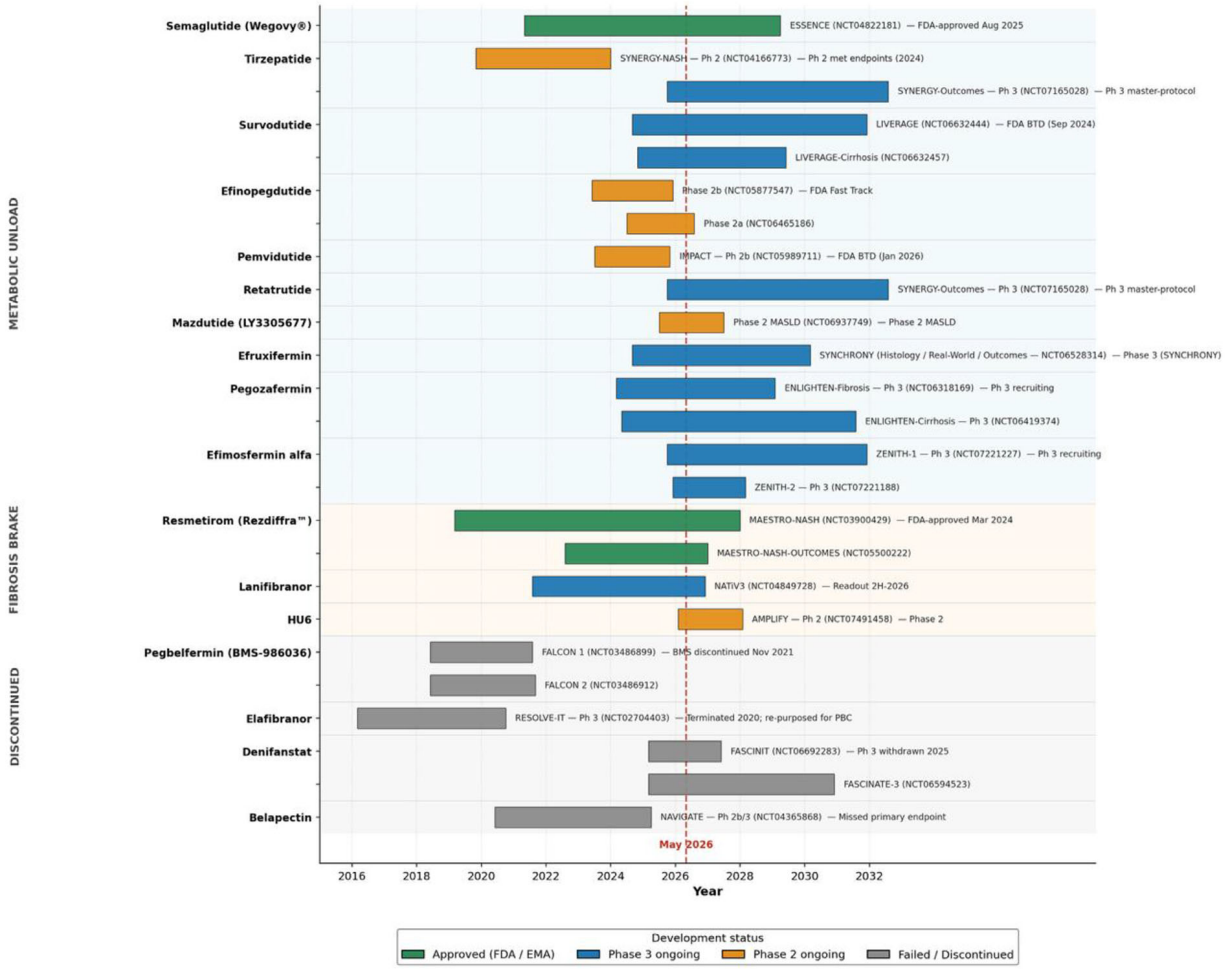
Development timeline of pharmacological therapies for MASLD/MASH across the clinical trial pipeline. Timeline illustrating the development and clinical trial progression of selected pharmacological therapies for metabolic dysfunction‐associated steatohepatitis (MASH), spanning the preclinical stage through Phase 13 clinical trials and regulatory approval. (Each bar = one pivotal trial; bar length = trial duration; colour = development status)

The rapidly evolving therapeutic landscape for MASLD/MASH can now be conceptualized through two mechanistic pillars: [1] metabolic unload, targeting the upstream drivers of steatosis and metabolic inflammation, and [2] fibrosis brake, acting downstream to halt or reverse fibrogenesis (Table 1). In practice, these pillars map to treatment intent: systemic cardiometabolic risk reduction and hepatic disease modification, respectively, with increasing convergence toward combination regimens in higher risk fibrosis. Given the rapidly expanding therapeutic landscape in MASLD/MASH, this review focuses primarily on a selected group of the most clinically advanced and promising therapies, whereas additional emerging agents and mechanisms are summarized in Table 1, Fig. 2.

**Table 1:** Development‐phase summary of pharmacological therapies for metabolic dysfunction‐associated steatotic liver disease (MASLD)/metabolic dysfunction‐associated steatohepatitis (MASH).

Mechanistic axis	Drug	Mechanism	Pivotal trial (NCT identifier)	Key clinical data	Trial start	Expected end/key readout	Development status	Regulatory/Status detail
**Metabolic unload**	**Semaglutide (Wegovy)**	GLP‐1 RA	ESSENCE (NCT04822181)	ESSENCE Phase 3: 63% MASH resolution, 37% fibrosis improvement at 72 weeks vs. 34%/22% placebo	May 2021	∼April 2029	**Approved**	FDA‐approved August 2025 for MASH F2–F3; Part 2 outcomes to 2029
	**Tirzepatide**	Dual GIP/GLP‐1 RA	SYNERGY‐NASH (Phase 2, NCT04166773)	SYNERGY‐NASH: 73% NASH resolution, ∼50% fibrosis improvement at 52 weeks	November 2019	January 2024	**Phase 2**	Phase 2 met endpoints (2024); Phase 3 master‐protocol recruiting
			SYNERGY‐Outcomes (Phase 3, NCT07165028)					
					October 2025	∼August 2032	**Phase 3 ongoing**	
	**Survodutide**	GLP‐1 + glucagon RA	LIVERAGE (NCT06632444)	Phase 2: 62% MASH improvement, 52% fibrosis regression at 48 weeks	September 2024	∼December 2031	**Phase 3 ongoing**	FDA Breakthrough Therapy designation (September 2024)
			LIVERAGE‐Cirrhosis (NCT06632457)		November 2024	∼June 2029	**Phase 3 ongoing**	
	**Efinopegdutide**	GLP‐1 + glucagon RA	Phase 2b (NCT05877547)	Phase 2a: 72.7% liver fat reduction, superior to semaglutide	June 2023	December 2025	**Phase 2b**	FDA Fast Track; Phase 2a superior to semaglutide for liver fat
			Phase 2a (NCT06465186)		July 2024	∼August 2026	**Phase 2a ongoing**	
	**Pemvidutide**	GLP‐1 + glucagon RA (1:1)	IMPACT (Phase 2b, NCT05989711)	Phase 2: 76% MRI‐PDFF reduction, significant ALT and cT1 improvement	July 2023	November 2025	**Phase 2b**	FDA Breakthrough Therapy designation (January 2026)
	**Retatrutide**	Triple agonist (GLP‐1/GIP/GCG)	SYNERGY‐Outcomes (NCT07165028)	Phase 2 obesity: 24% weight loss, 60%–80% MRI‐PDFF reduction; MASH trial ongoing	October 2025	∼August 2032	**Phase 3 ongoing**	Phase 3 master‐protocol recruiting
	**Mazdutide (LY3305677)**	GLP‐1/Glucagon RA	Phase 2 MASLD (NCT06937749)	AASLD 2025 (#5002): >60% steatosis reduction and ALT improvement	July 2025	∼July 2027	**Phase 2 ongoing**	Phase 2 MASLD
	**Efruxifermin**	FGF21 analog	SYNCHRONY Histology, Real‐World and Outcomes (NCT06528314)	HARMONY/SYMMETRY: 41% NASH resolution + fibrosis improvement, cirrhosis reversal at 96 weeks	September 2024	∼March 2030	**Phase 3 ongoing**	Phase 3 (SYNCHRONY)
	**Pegozafermin**	FGF21 analog	ENLIGHTEN‐Fibrosis (Phase 3, NCT06318169)	ENLIVEN: 27% fibrosis improvement vs. 7% placebo; MR‐PDFF 60%–70%	March 2024	∼February 2029	**Phase 3 ongoing**	Phase 3 ongoing and recruiting
			ENLIGHTEN‐Cirrhosis (Phase 3, NCT06419374)		May 2024	∼August 2031	**Phase 3 ongoing**	
	**Efimosfermin alfa**	Long‐acting FGF21 analog	ZENITH‐1 (NCT07221227)	AASLD 2025 (#5011): durable fat and fibrosis biomarker improvements to 48 weeks	October 2025	∼December 2031	**Phase 3 ongoing**	Phase 3 ongoing and recruiting
			ZENITH‐2 (NCT07221188)		December 2025	∼March 2028	**Phase 3 ongoing**	
**Fibrosis Brake**	**Resmetirom (Rezdiffra)**	THR‐β agonist	MAESTRO‐NASH (NCT03900429)	MAESTRO‐NASH: 26%–30% NASH resolution, 24%–26% fibrosis improvement; MAESTRO‐OLE shows 2‐year durability	March 2019	∼January 2028	**Approved**	FDA‐approved (2024) F2–F3
			MAESTRO‐NASH‐OUTCOMES (NCT05500222)	August 2022	∼January 2027			
	**Lanifibranor**	Pan‐PPAR (α/δ/γ)	NATiV3 (NCT04849728)	NATIVE: significant NAS reduction, 49% NASH resolution; fibrosis signal improving	August 2021	∼December 2026	**Phase 3 ongoing**	Phase 3 NATiV3 (readout 2026)
	**HU6**	Controlled metabolic accelerator (mitochondrial uncoupler)	AMPLIFY (Phase 2, NCT07491458)	Phase 2: significant PDFF reduction with minimal weight loss; improved visceral fat	February 2026	∼February 2028	**Phase 2 ongoing**	Phase 2
**Discontinued**	**Pegbelfermin (BMS‐986036)**	FGF21 analog	FALCON 1 (NCT03486899)		June 2018	∼August 2021	**Failed/Discontinued**	Both Phase 2b studies failed; BMS discontinued (November 2021)
			FALCON 2 (NCT03486912)		June 2018	∼September 2021		
	**Elafibranor**	Dual PPAR‐α/δ	RESOLVE‐IT (Phase 3) (NCT02704403)		March 2016	∼October 2020	**Failed/Discontinued**	Phase 3 terminated for MASH (Genfit, 2020); now repurposed for PBC
	**Denifanstat**	FASN inhibitor	FASCINIT (NCT06692283)		March 2025	∼June 2027	**Failed/Discontinued**	Phase 3 trials withdrawn 2025 (business/funding decision); future MASH program paused
			FASCINATE‐3 (NCT06594523)		March 2025	∼December 2030		
	**Belapectin**	Galectin‐3 inhibitor	NAVIGATE (Phase 2b/3, NCT04365868)		June 2020	April 2025	**Failed/Discontinued**	Missed composite primary endpoint; per‐protocol benefit only; further development under review

Abbreviation: PPAR, peroxisome proliferator‐activated receptor.

### Metabolic unload: can we switch off the engine?

The metabolic unloading therapeutic class encompasses incretin‐based agents and fibroblast growth factor (FGF) analogs, which target the upstream metabolic drivers of MASLD by reducing hepatic lipid influx, de novo lipogenesis, and lipotoxic stress [35]. Through sustained reductions in liver fat, these agents indirectly attenuate hepatocellular inflammation, oxidative injury, and downstream fibrogenic signaling. In parallel, they exert systemic metabolic benefits, including improvements in insulin sensitivity, adipose tissue function, and atherogenic dyslipidemia, which are particularly relevant given the high prevalence of Type 2 diabetes and cardiometabolic disease in MASLD populations. This class includes GLP‐1 receptor agonists, dual and triple incretins, and FGF21 analogs, which are increasingly viewed as foundational components of combination strategies aimed at modifying disease trajectory rather than treating isolated hepatic endpoints. SGLT2 inhibitors are also relevant as background metabolic therapy, with consistent reductions in liver fat and enzymes, although robust histology data in MASH remain more limited than for incretin and FGF21 programs.

#### GLP‐1 receptor agonists (incretin‐based therapies)

GLP‐1 receptor agonists (GLP‐1 RAs) are established treatments for Type 2 diabetes and obesity, and they have significant relevance in MASLD due to their weight loss and insulin‐sensitizing effects [36]. The liver benefits of GLP‐1 RAs were first recognized in patients with Type 2 diabetes, where treatment was associated with improvements in liver enzymes and reductions in hepatic steatosis on imaging [37]. Now, GLP‐1 analogs are being specifically evaluated for MASH. Across trials, the magnitude of weight loss is a major mediator of histological response, but liver benefits are not fully explained by weight change alone, supporting their positioning as cornerstone metabolic therapies in MASLD.

##### Semaglutide

A once‐weekly GLP‐1 RA already approved for T2D and obesity, semaglutide was one of the first incretin therapies tested in MASH. On August 15, 2025, the US FDA approved semaglutide (Wegovy) for the treatment of MASH in adults with moderate‐to‐advanced fibrosis. The approval was supported by data from the Phase 3 ESSENCE trial, in which semaglutide achieved MASH resolution in 63% of treated patients without worsening of liver fibrosis compared to 34% of participants receiving placebo (*p* < 0.001), and 37% of the semaglutide group experienced a reduction in liver fibrosis without worsening steatohepatitis, versus 22% in the placebo group (*p* < 0.001) [25]. Patients treated with semaglutide also experienced significant weight loss, with a mean change of −10.5% compared to −2.0% in the placebo group. This planned interim analysis at Week 72 based on liver biopsy results included 800 clinical trial participants randomly assigned to either semaglutide (534 participants) or placebo (266 participants) in addition to lifestyle changes. Participants were on stable doses of lipid‐lowering, glucose‐management, and weight‐loss medications.

The trial will continue for a total of 240 weeks to determine whether inflammation and scarring improvements seen after 72 weeks translate into decreases in death, liver transplant, and other liver‐related events [38]. This long‐term follow‐up is critical because regulatory and guideline pathways are increasingly anchored to clinically meaningful outcomes rather than histology alone.

The treatment was generally well‐tolerated, with gastrointestinal adverse events such as nausea and diarrhea being more common in the semaglutide group. However, no new safety signals were identified during the trial.

*Other GLP‐1s*: Previously, liraglutide (daily GLP‐1 RA) showed a modest benefit in the LEAN trial with 39% NASH resolution versus 9% placebo, but again no significant fibrosis change [39]. Liraglutide's use is limited by GI side effects and daily injections. These data established proof of concept for incretin‐mediated steatohepatitis resolution but highlighted the need for greater potency and durability to shift fibrosis.

##### Incretin co‐agonists and triple agonists

Building on GLP‐1 success, multi‐agonist peptides that activate multiple gut hormone receptors, for example, GIP, glucagon along with GLP‐1 are in development. These aim to enhance weight loss and metabolic effects, which could translate to greater MASH improvements. The last 2 years have brought breakthrough data for this class. Multi‐agonists also raise a practical treatments question: How to balance efficacy against gastrointestinal tolerability and longer term discontinuation in real‐world populations with multimorbidity.

Tirzepatide (dual GIP and GLP‐1 receptor agonist): Tirzepatide (already approved for T2D and in trials for obesity) was assessed in the Phase 2b SYNERGY‐NASH trial. Results presented at EASL Congress 2024 and published in *NEJM* highlighted that at 52 weeks, 73% of patients on tirzepatide 15 mg weekly achieved MASH resolution with no worsening of fibrosis, versus 13% on placebo. Even at the lowest dose (5 mg), 52% achieved this endpoint. Additionally, fibrosis improvement (≥1 stage) was seen in more than half of patients at all tirzepatide doses (∼51%–54%), compared to 33% in placebo. These are among the highest response rates reported in MASH in a 1‐year trial. Tirzepatide also induced profound weight loss (10%–15% body weight) and improved all metabolic parameters (glycemic control, lipids). Importantly, histological benefits appeared to correlate with the magnitude of weight reduction. Safety in MASH patients was similar to obesity trials: mainly gastrointestinal side effects (nausea, vomiting) during dose escalation, with low rates of discontinuation [40]. *Status*: Phase 3 for tirzepatide in MASH is being initiated, aiming to confirm histological efficacy and eventually outcomes. Given its dual metabolic action, tirzepatide might become a cornerstone for patients with MASLD and obesity or diabetes. Notably, it could be complementary to liver‐directed drugs: for example, one can envision combining tirzepatide for metabolic correction with an anti‐fibrotic (like an FGF21 analog) for optimal results [40]. Positioning: Tirzepatide is best viewed as high‐potency metabolic backbone therapy, with the major remaining uncertainty being long‐term outcomes and durability of fibrosis regression.

Survodutide (BI 456906; dual GLP‐1 and glucagon receptor agonist): Survodutide is another once‐weekly injectable co‐agonist, differing from tirzepatide in that it stimulates the glucagon receptor that can increase energy expenditure and reduce liver fat instead of GIP agonism. This glucagon‐mediated pathway may enhance hepatic fat mobilization, increase energy expenditure, and further improve metabolic dysfunction, making it particularly attractive for MASLD/MASH. In a Phase 2 trial presented at EASL 2024, survodutide demonstrated substantial histological efficacy in patients with MASH. After 48 weeks of treatment, up to 62% of patients (at 4.8 mg dose) had improvement in MASH (resolution of steatohepatitis without fibrosis worsening) versus 14% on placebo. Fibrosis improvement ≥1 stage occurred in 52% of treated patients versus 26% placebo [41]. An earlier top‐line release noted 83% of patients on a high dose achieved an NAFLD activity score reduction (≥2 points), indicating broad histologic improvement. Survodutide also met all secondary endpoints, including statistically significant improvement in fibrosis stage distribution and a large reduction in liver fat content. Patients also lost substantial weight during the trial.

*Status*: Survodutide has now progressed into Phase 3 development for MASH. The pivotal LIVERAGE trial is actively recruiting adults with biopsy‐confirmed MASH and F2–F3 fibrosis, whereas LIVERAGE‐Cirrhosis is evaluating survodutide in compensated MASH cirrhosis. In parallel, the broader Phase 3 obesity program is ongoing, with SYNCHRONIZE‐1 reporting significant weight loss in 2026. Survodutide's differentiation is the glucagon activity, which can sometimes cause mild elevations in transaminases or blood pressure, hence requiring monitoring. But like tirzepatide, it holds promise as a powerful agent addressing the metabolic root causes of MASH. Glucagon receptor activity may widen suitability for patients where maximal liver fat reduction is prioritized but may require more structured monitoring in routine care.

Efinopegdutide (formerly MK‐6024 or HM12525A; dual GLP‐1/glucagon agonist): Efinopegdutide is another dual agonist with an Fc component (a portion of an antibody protein used to prolong the drug's half‐life and enable once‐weekly dosing). A Phase 2a trial (24 weeks) comparing efinopegdutide to semaglutide was published in 2023 [42]. Importantly, the comparator arm used semaglutide 1.0 mg weekly rather than the currently approved 2.4 mg dose used in obesity and emerging MASH practice, which should be considered when interpreting comparative efficacy. Efinopegdutide led to a 72.7% relative reduction in liver fat content, significantly greater than the 42.3% reduction with semaglutide at the same time point. This suggests adding glucagon activity enhances clearance of liver fat. The FDA granted Fast Track designation to efinopegdutide in MASH. Further trials will evaluate histological endpoints. Liver fat reduction is a useful early signal, but translation to fibrosis endpoints remains the key question for positioning in treatment algorithms.

Pemvidutide (ALT‐801; dual GLP‐1/glucagon): Pemvidutide is engineered to provide balanced co‐activation of GLP‐1 and glucagon pathways and incorporates a peptide sequence designed to slow systemic absorption, thereby improving gastrointestinal tolerability [43]. On January 5, 2026, the US FDA granted Breakthrough Therapy Designation (BTD) for pemvidutide for the treatment of patients with MASH. In December 2025, 48‐week top‐line IMPACT data were reported showing that continued treatment with pemvidutide resulted in statistically significant improvements versus placebo in key NITs, including ELF and LSM, with additional reductions from Week 24 across both dose levels, supporting ongoing anti‐fibrotic activity. At 48 weeks, patients receiving the 1.8 mg dose achieved further weight loss with no evidence of plateauing, and pemvidutide maintained the favorable tolerability profile observed at 24 weeks, including a lower discontinuation rate due to adverse events compared with placebo. As this is currently noninvasive endpoint‐based confirmation, with histology and outcomes will determine the strength of any disease‐modifying claim.

##### Triple agonists (GLP‐1/GIP/glucagon)

The next frontier is triple agonists like **retatrutide** (LY3437943). SYNERGY‐Outcomes is an ongoing Phase 3 study designed to simultaneously assess retatrutide and tirzepatide treatment for the prevention of major adverse liver outcomes (MALO) in adults with high‐risk MASLD. Retatrutide has shown in Phase 2 studies for obesity unparalleled weight loss (24% at 48 weeks in obesity trial) and, notably, marked reductions in liver fat (MRI‐PDFF ↓ 60%–80% depending on dose) [44]. Importantly, the Phase 3 MASLD program represents a significant shift in trial design within hepatology, as enrollment is increasingly based on noninvasive fibrosis assessment rather than mandatory liver biopsy alone, reflecting the growing acceptance of sequential noninvasive testing pathways in both clinical practice and therapeutic trials. Furthermore, SYNERGY‐Outcomes is designed as a long‐term, outcome‐driven study focused on clinically meaningful endpoints, including progression to cirrhosis, hepatic decompensation, liver transplantation, hepatocellular carcinoma, and liver‐related mortality, rather than relying solely on short‐term histological improvement. The triple agonist concept could further amplify metabolic benefits, though tolerability, especially GI side effects, needs careful management due to potent anorexic effects. If outcome trials are positive, these agents may redefine first‐line therapy for high‐risk MASLD by combining weight loss, metabolic control, and event reduction.

Summary: Incretin‐based therapies, especially GLP‐1 analogs and their dual/triple cousins, have emerged by 2026 as transformative therapeutic options for patients with MASLD and associated metabolic dysfunction. They improve obesity and insulin resistance that drive MASH, achieving histological resolution of steatohepatitis at rates previously unseen. Thus, combination therapies (GLP‐1 + anti‐fibrotic) are a logical next step. Nonetheless, given their safety profile and extra‐hepatic benefits (cardiovascular risk reduction demonstrated in trials for diabetes/obesity), GLP‐1 RAs and related drugs are likely to be integral in future MASLD management treating the patient's whole metabolic disease, not just the liver. A practical implication is that for many patients with MASLD and obesity or diabetes, an incretin‐based agent may be an appropriate first pharmacological step irrespective of whether a liver‐specific drug is added later based on fibrosis risk.

#### Fibroblast growth factor analogs (FGF‐based therapies)

FGFs represent a broad family of multifunctional proteins with essential roles in development, organ formation, and metabolic homeostasis. Some representatives of the family include FGF19 and FGF21, which are two hormones that play significant roles in metabolic regulation and liver health. FGF19 is mostly expressed in the ileal enterocytes and is released into the enterohepatic circulation in response to bile acids signaling. It inhibits hepatic bile acid synthesis and has anti‐steatotic, anti‐inflammatory, and anti‐fibrotic effects. FGF21, on the other hand, is highly expressed in the liver and is released in response to high glucose, high free‐fatty acids, and low amino‐acid supply. It regulates energy, glucose, and lipid homeostasis by actions in the CNS and in adipose tissue. Both hormones have been shown to improve metabolic profiles, reduce liver fat, and have anti‐fibrotic and anti‐inflammatory actions [45]. Native FGF19/21 has short half‐lives, so analogs have been engineered for pharmacotherapy in MASLD. Clinically, the appeal of FGF21 analogs is that they may deliver fibrosis regression that is not solely dependent on large‐magnitude weight loss, making them attractive partners for incretin‐based backbones.

##### Efruxifermin (EFX, AKR‐001) *FGF21 analog*

Mechanism: Efruxifermin is a long‐acting Fc‐FGF21 fusion protein that activates FGF21 receptors involved in glycemic control, lipid oxidation, and energy expenditure. It powerfully reduces hepatic steatosis and can directly impact fibrosis via effects on stellate cells and inflammation [46].

Efficacy: Efruxifermin has delivered some of the most impressive histological results to date. In a Phase 2a trial (BALANCED), 16‐week treatment led to marked reductions in liver fat and significant NASH resolution rates versus placebo [46]. Building on that, the Phase 2b HARMONY trial in patients with F2–F3 fibrosis showed high efficacy where at 24 weeks, 41% of patients on efruxifermin (50 mg weekly) achieved NASH resolution with fibrosis improvement, versus 2% on placebo. Fibrosis regression (≥1 stage improvement) occurred in 39% on treatment versus 9% placebo [47]. These short‐term histology outcomes were among the best seen in MASH to date.

96‐week outcomes: Importantly, efruxifermin appears to induce deeper fibrosis reversal with longer therapy. Data from the open‐label extension of HARMONY up to 96 weeks, presented at AASLD 2024, showed near‐complete histological disease reversal in many patients. AASLD late‐breaker findings reported that the proportion of “at‐risk” MASH patients (fibrosis F2–F3) was significantly reduced with efruxifermin, and some patients achieved resolution of NASH and regression of fibrosis to F0 essentially histologically normal livers [48].

Cirrhosis (F4) cohort—SYMMETRY: Efruxifermin is the first drug to show potential to reverse cirrhosis due to MASH. In the Phase 2b SYMMETRY trial for compensated cirrhosis (F4), 96‐week interim data presented at EASL 2025 showed that approximately 39% of patients achieved fibrosis improvement from F4 to F3 or lower without worsening of MASH, whereas nearly 29% demonstrated complete histological reversal of cirrhosis [49]. Noninvasive markers of fibrosis and metabolic health (liver stiffness, ELF, HbA1c, lipids) also improved. Investigators hailed this as a “pivotal moment,” with efruxifermin becoming the first drug ever to demonstrate reversal of MASH cirrhosis in a clinical trial. These findings, though in a mid‐stage trial, raise hope that advanced fibrosis may be reversible with prolonged therapy.

Safety: EFX is generally well‐tolerated. The main side effects are GI (mild diarrhea, nausea) in some patients, typically transient. There were no significant safety concerns in 96‐week data aside from expected reversible GI events and some injection site reactions. FGF21 analogs can cause weight loss; efruxifermin patients lost a modest ∼2–5 kg on average, which is a beneficial metabolic effect [48].

Status: Phase 3 ongoing. Three Phase 3 trials (the SYNCHRONY program) in various MASH populations have been initiated: pre‐cirrhotic F2–F3 (with histology endpoint), compensated cirrhosis, and a long‐term outcomes trial. Efruxifermin has Fast Track designation. Given the strong Phase 2 results, it is regarded as a leading candidate that could become the first therapy to truly reverse advanced fibrosis. Earliest potential FDA submission is in 2026 if Phase 3 histology endpoints are met.

##### Pegozafermin (BIO89‐100) *FGF21 analog*

Pegozafermin is a glycoPEGylated FGF21 analog with an extended half‐life (weekly or biweekly dosing). In the Phase 2b ENLIVEN trial (24 weeks) in NASH F2–F3, pegozafermin showed dose‐dependent benefits. Both weekly and Q2W dosing met the primary endpoint of NASH improvement: pooled analysis showed 27% of treated patients had ≥1‐stage fibrosis improvement without NASH worsening versus 7% on placebo (*p* < 0.001). NASH resolution rates were also higher with pegozafermin (26%–27% vs. 7% placebo). These results were published in *NEJM* [50] and represent significant histological efficacy at 6 months. Pegozafermin also reduced liver fat by 60%–70% (MRI‐PDFF) and improved metabolic markers (ALT, triglycerides, HOMA‐IR).

Safety: Pegozafermin was well‐tolerated; adverse effects included mild injection site reactions and GI symptoms. There was minimal incidence of pruritus and no concerning safety signals.

Status: With positive Phase 2b data, 89bio has initiated a global Phase 3 program. This comprises ENLIGHTEN‐Fibrosis, enrolling patients with biopsy‐confirmed MASH and stage F2–F3 fibrosis, and ENLIGHTEN‐Cirrhosis, targeting compensated MASH‐related cirrhosis (F4), both designed to support regulatory approval based on histologic endpoints. In parallel, pegozafermin is being evaluated in a Phase 3 trial for severe hypertriglyceridemia. The program has received FDA BTD and EMA PRIME eligibility, underscoring the unmet clinical need and therapeutic promise of FGF21‐based approaches in advanced metabolic liver disease. As of early 2026, Phase 3 trials are ongoing, with key histology‐based readouts anticipated from 2027 onward. Positioning: The consistent MRI PDFF and histology signals make pegozafermin a leading candidate for combination regimens, particularly where triglycerides and insulin resistance are prominent.

##### Aldafermin (NGM‐282) FGF19 analog

Aldafermin, an analog of the enteric hormone FGF19, showed early promise by reducing liver fat and fibrosis in Phase 2a studies [51]. A 24‐week trial in NASH F2–F3 demonstrated significant reduction in fibrosis (38% of patients improved ≥1 stage on aldafermin vs. 18% placebo) and NASH activity. However, a larger Phase 2b (ALPINE 2/3) did not meet its primary endpoint of fibrosis improvement, and development in NASH was halted in 2021 [52].

##### Pegbelfermin (BMS‐986036) *FGF21 analog*

Pegbelfermin (BMS‐986036) was among the first engineered FGF21 analogs designed to overcome the short half‐life of native FGF21 and was evaluated in multiple Phase 2 clinical trials in NASH/MASLD and obesity/Type 2 diabetes populations. Early studies demonstrated that 16 weeks of pegbelfermin significantly reduced hepatic fat content measured by MRI‐PDFF compared with placebo [53]. However, the program did not progress into successful pivotal histology‐improving outcomes: larger Phase 2b studies (FALCON 1 and FALCON 2) failed to meet primary histopathologic endpoints, despite showing improvements in noninvasive measures of steatosis, fibrosis, and inflammation, leading to discontinuing further development of pegbelfermin in MASH [54].

Summary: FGF‐based therapies (especially FGF21 analogs) are emerging as some of the most potent agents for both metabolic improvement and fibrosis regression. Advanced clinical candidates such as Efruxifermin and Pegozafermin have demonstrated robust histological efficacy in Phase 2 and early Phase 3 programs, including high rates of steatohepatitis resolution and clinically meaningful fibrosis improvement, with signals of fibrosis regression over relatively short treatment durations. In parallel, these agents consistently improve cardiometabolic parameters, including reductions in liver fat, triglycerides, body weight, and insulin resistance, which is an important advantage in a population in which approximately half of patients have Type 2 diabetes. As injectable biologics, long‐term adoption will depend on durability of histologic benefit, cardiovascular safety, and real‐world tolerability; however, regulatory momentum and ongoing pivotal trials suggest that FGF21 analogs are now among the leading late‐stage contenders to become foundational therapies for MASLD. By early 2026, the field is increasingly converging on combination strategies, with FGF21 analogs viewed as strong backbone agents to be paired with complementary mechanisms such as GLP‐1 receptor agonists, dual/triple incretins, or thyroid hormone receptor‐β agonists to address the multifactorial metabolic and fibrotic drivers of disease. In treatment terms, the key differentiator will be whether fibrosis regression is reproducible, durable, and additive to incretin‐mediated metabolic unloading.

### Fibrosis brake: how do we halt or reverse scar? THR‐β agonists, PPARs, and anti‐fibrotic strategies

Fibrosis stage remains the single strongest determinant of liver‐related and overall mortality in MASLD, underscoring the urgent need for therapies that can not only slow progression but actively promote fibrosis regression. Historically, anti‐fibrotic drug development in steatohepatitis has been disappointing, in part because fibrosis is a dynamic, multicellular process driven by persistent metabolic injury, inflammation, and maladaptive wound healing rather than a single molecular pathway. Recent advances, however, indicate that fibrosis may be more biologically reversible than previously appreciated, particularly when upstream metabolic stress is effectively corrected. This has shifted the treatment paradigm toward pairing metabolic backbone therapy with agents that deliver incremental anti‐fibrotic efficacy in patients with established F2–F3 disease and, ultimately, in compensated cirrhosis.

Liver‐directed metabolic agents have emerged as a credible means of applying a fibrosis brake. Selective thyroid hormone receptor‐β agonists, exemplified by Resmetirom, reduce hepatocellular lipotoxicity and oxidative stress, leading to downstream attenuation of stellate cell activation and reproducible improvements in fibrosis in non‐cirrhotic MASH. In parallel, next‐generation PPAR agonists, particularly pan‐PPAR compounds such as lanifibranor, target multiple nodes of disease biology, combining improvements in insulin resistance and dyslipidemia with direct anti‐inflammatory and anti‐fibrotic effects at the cellular and extracellular matrix level.

This section examines the mechanistic basis and clinical evidence supporting these therapies and discusses how they may be used alone or in rational combinations to meaningfully alter the natural history of MASLD.

#### Thyroid hormone receptor‐β agonist: resmetirom (MGL‐3196)

- Mechanism: Resmetirom is an orally administered, liver‐directed thyroid hormone receptor‐β (THR‐β) agonist that enhances hepatic lipid handling (including fatty‐acid oxidation) and reduces intrahepatic lipotoxic burden, producing consistent downstream improvements in steatosis and atherogenic lipids (notably LDL‐C). As a liver‐selective nuclear receptor modulator, resmetirom primarily targets hepatic fat and lipid metabolism rather than driving large‐magnitude weight loss, which supports its role as a liver‐directed disease‐modifying option in patients with established F2–F3 fibrosis.
- Efficacy data: In the Phase 3 MAESTRO‐NASH trial (52‐week biopsy analysis), both 80 and 100 mg doses met the FDA‐specified histologic primary endpoints: MASH/NASH resolution with no worsening of fibrosis occurred in 26% (80 mg) and 30% (100 mg) versus 10% with placebo; and ≥1‐stage fibrosis improvement with no worsening of disease activity occurred in 24% (80 mg) and 26% (100 mg) versus 14% with placebo [55]. Resmetirom also improved secondary measures, including LDL‐C and noninvasive liver fat metrics [32]. These effect sizes are clinically meaningful because they show concurrent improvement in steatohepatitis activity and fibrosis in a pre‐cirrhotic population, with a parallel reduction in atherogenic lipids that may be advantageous in a group with high baseline cardiovascular risk.
- Safety: Across MAESTRO‐NASH and regulatory reviews, resmetirom was generally well tolerated; the most frequent adverse events were gastrointestinal (e.g., diarrhea/nausea) and thyroid‐axis laboratory changes consistent with target engagement, supporting routine thyroid monitoring in clinical use. In practice, baseline thyroid history and thyroid function tests are helpful before initiation, with follow‐up testing to ensure biochemical stability.
- Regulatory status: Resmetirom received FDA accelerated approval on 14 March 2024 for adults with noncirrhotic MASH/NASH with moderate‐to‐advanced fibrosis (F2–F3), to be used with diet and exercise; approval is contingent on confirmatory outcomes evidence. In Europe, resmetirom subsequently received EU conditional marketing authorization (August 18, 2025), with an EMA EPAR now published. These approvals position resmetirom as the first broadly available liver‐specific pharmacotherapy for MASH with F2–F3 fibrosis, while reinforcing the need for outcomes confirmation and careful patient selection.
- Guideline integration: The AASLD October 2024 updates provide an implementation framework that aligns patient selection with evidence of F2–F3 disease (often via noninvasive risk stratification pathways in routine practice) and recommend monitoring of thyroid function and liver tests during therapy. Operationally, this favors integration into sequential noninvasive testing pathways that identify likely F2–F3 patients and avoid treatment of low‐risk steatosis alone.
- Ongoing confirmatory/outcomes trials: The Phase 3 MAESTRO‐NASH‐OUTCOMES program is evaluating whether resmetirom modifies clinically meaningful endpoints (liver‐related outcomes) in advanced disease populations, providing the key confirmatory evidence expected by regulators for conversion beyond accelerated/conditional pathways. For a treatment review, this is the key remaining uncertainty: Whether histology improvements translate into fewer clinical events and durable benefit with longer term use.
- Positioning: Resmetirom is best viewed as a liver‐directed fibrosis brake option for biopsy‐proven or high‐confidence F2–F3 disease, particularly when weight loss‐based therapies are insufficient, not tolerated, or contraindicated, and it may be additive to metabolic backbone therapy.

#### PPAR agonists (peroxisome proliferator‐activated receptors)

The PPARs (α, δ/β, and γ) are nuclear transcription factors that regulate lipid metabolism, insulin sensitivity, inflammation, and fibrogenesis, making them attractive targets for the multifactorial pathogenesis of MASLD/MASH [56]. Although earlier single or dual isoform agonists delivered mixed histological results, renewed interest has been driven by next‐generation pan‐PPAR modulation, aiming to balance metabolic efficacy with acceptable tolerability. In treatment terms, the central question is whether broad PPAR activation can deliver reproducible anti‐fibrotic benefit without trade‐offs that limit real‐world uptake, particularly weight gain, fluid retention, and hematologic effects.

##### Lanifibranor

Lanifibranor, an oral pan‐PPAR (α/δ/γ) agonist developed by Inventiva, remains the most advanced and promising agent in this class. In the Phase 2b NATIVE trial, lanifibranor (1200 mg daily) met its primary endpoint at 24 weeks, achieving a significantly greater proportion of patients with a ≥2‐point reduction in SAF activity score without worsening of fibrosis versus placebo. Post hoc analyses demonstrated high rates of steatohepatitis resolution and signals of fibrosis improvement, alongside favorable effects on noninvasive fibrogenesis markers (including collagen turnover biomarkers), liver enzymes, and lipid parameters [57]. These data supported FDA Breakthrough Therapy and EMA PRIME designations. For clarity, the NATIVE endpoint was activity improvement without fibrosis worsening, with fibrosis outcomes supportive but not the primary driver of the headline result, which matters when comparing across late‐stage agents that are increasingly judged on fibrosis improvement.

From a metabolic perspective, lanifibranor improves insulin sensitivity and glycemic control through PPARγ activation, with only modest weight gain (1–2 kg). More recently, results from the Phase 2b LEGEND study in patients with MASH and Type 2 diabetes, presented in 2025, showed that lanifibranor alone or in combination with the SGLT2 inhibitor empagliflozin met its primary endpoint of HbA1c reduction and improved multiple liver‐related biomarkers [58]. This supports potential use in patients with prominent insulin resistance where an oral agent is preferred, although liver biomarker improvements still require Phase 3 histology confirmation for regulatory positioning in MASH.

Lanifibranor is currently being evaluated in the large global Phase 3 NATiV3 trial (1200 patients with F2–F3 MASH), assessing 72‐week histological endpoints with longer term clinical follow‐up. Enrolment was completed in early 2025, with top‐line histology data anticipated in 2026–2027. If positive, lanifibranor could seek accelerated approval based on histological endpoints, positioning it as the first oral, single‐agent therapy combining metabolic and anti‐fibrotic mechanisms.

Safety data to date indicate good overall tolerability, with mild anemia (consistent with plasma‐volume expansion) and modest weight gain observed. In practice, these effects may be particularly relevant in patients with heart failure risk, advanced chronic kidney disease, or baseline anemia and will influence selection if multiple effective options are available. Importantly, combination therapy with the SGLT2 inhibitor empagliflozin in the LEGEND study appeared to partially mitigate some of the fluid‐retention and weight‐related effects typically associated with PPARγ activation, likely through the natriuretic and osmotic diuretic properties of SGLT2 inhibition. Although the study was not specifically powered for edema‐related endpoints, these findings raise the possibility that combination approaches may improve the metabolic and tolerability profile of lanifibranor in clinical practice [58].

##### Elafibranor

Elafibranor, a dual PPARα/δ agonist developed by Genfit, failed to meet its primary endpoint in the Phase 3 RESOLVE‐IT trial and was discontinued for MASH in 2020 [59]. Although modest metabolic and anti‐inflammatory effects were observed, histological efficacy was insufficient. Development has since pivoted to cholestatic liver disease (PBC), highlighting the limitations of partial PPAR modulation in steatohepatitis. This negative program is important context because it set a higher bar for later PPAR agents and reinforced that biomarker improvements alone do not reliably translate into fibrosis benefit.

##### Pioglitazone

Pioglitazone, a PPARγ agonist widely used in Type 2 diabetes, has consistently demonstrated steatohepatitis resolution and some fibrosis improvement in biopsy‐proven MASH, particularly in patients with diabetes. However, weight gain, fluid retention, fracture risk, and concerns regarding long‐term safety have constrained uptake. Although current guidelines still endorse pioglitazone as an option in selected patients, its limitations underscore the need for more balanced PPAR strategies, exemplified by lanifibranor. In a treatment review, pioglitazone is best framed as an accessible metabolic option with liver benefits in carefully selected patients, rather than a dedicated anti‐fibrotic strategy.

#### Other PPAR‐targeting agents

Other agents, including saroglitazar (dual PPARα/γ, approved in India based on surrogate endpoints) and pemafibrate (selective PPARα agonist), have shown improvements in liver enzymes and lipid parameters but lack convincing histological efficacy in MASH. Notably, pemafibrate failed to demonstrate cardiovascular benefit in the PROMINENT trial and did not improve liver histology, reinforcing the view that isolated PPARα agonism is insufficient for disease modification [60]. This further supports a shift toward multi‐pathway approaches or combination regimens when fibrosis modification is the goal.

Overall, PPAR agonists remain biologically compelling due to their ability to address insulin resistance, dyslipidemia, inflammation, and fibrogenesis. Among them, lanifibranor stands out as the leading next‐generation candidate, with the potential to deliver clinically meaningful histological benefit as an oral, multi‐mechanistic therapy. If Phase 3 results are positive, lanifibranor could occupy a key position in the MASLD treatment algorithm either as monotherapy in patients unsuitable for injectables or as part of rational combination regimens with incretin‐based agents or other liver‐directed therapies. A practical differentiator will be whether lanifibranor offers additive fibrosis benefit on top of incretin‐based metabolic unloading while maintaining tolerability that supports long‐term adherence.

#### Future directions: combination therapy and precision treatment strategies

As the therapeutic landscape for MASLD/MASH rapidly evolves, combination therapy is increasingly expected to become a central treatment strategy, similar to existing treatment models in Type 2 diabetes, oncology, and viral hepatitis management [61, 62]. Current pharmacological agents demonstrate differing therapeutic strengths: Incretin‐based therapies primarily achieve substantial weight loss and steatohepatitis resolution, whereas emerging anti‐fibrotic agents may exert greater effects on fibrosis regression and long‐term liver‐related outcomes. Consequently, future management is likely to involve rational combinations targeting complementary pathogenic pathways, including insulin resistance, lipotoxicity, inflammation, mitochondrial dysfunction, and fibrogenesis simultaneously [61, 63].

Several combination approaches are already emerging in clinical development. GLP‐1 receptor agonists and dual/triple incretin agonists may ultimately be combined with anti‐fibrotic therapies such as lanifibranor, efruxifermin, resmetirom, or survodutide to optimize both metabolic and fibrosis‐related endpoints [64]. Similarly, combining PPAR agonists with SGLT2 inhibitors may improve insulin resistance while mitigating adverse effects such as fluid retention or weight gain associated with PPARγ activation [65]. Early data from the LEGEND study suggest that empagliflozin may partially offset some tolerability limitations of lanifibranor while preserving metabolic efficacy [58].

Recent retrospective data suggest that combination therapy with GLP‐1 receptor agonists and SGLT2 inhibitors in MASLD is associated with significantly lower risks of all‐cause hospitalization, mortality, major adverse cardiovascular, kidney, and liver outcomes compared with SGLT2 inhibitor therapy alone, supporting the potential value of multi‐target metabolic treatment strategies [66].

Sequential treatment strategies may also emerge, whereby potent weight‐loss therapies are introduced early to reduce hepatic steatosis and systemic metabolic burden, followed by fibrosis‐directed therapies in patients with persistent advanced disease.

Precision stratification according to disease phenotype and fibrosis stage will likely become increasingly important in future MASLD care pathways [66]. Patients with obesity and Type 2 diabetes may derive greatest benefit from incretin‐based therapies because of their profound effects on weight reduction, glycemic control, and cardiovascular risk. In contrast, patients with advanced fibrosis or compensated cirrhosis may require therapies with stronger anti‐fibrotic activity and long‐term outcome‐based evidence. Agents such as efruxifermin and survodutide are particularly promising in advanced disease given emerging data suggesting regression of severe fibrosis and potential reduction in major adverse liver outcomes (MALO) [36, 37]. Conversely, individuals with earlier stage disease and predominant metabolic dysfunction may benefit most from aggressive metabolic therapy combined with structured lifestyle intervention.

Noninvasive testing is also likely to play an increasingly important role in treatment selection and monitoring. Sequential algorithms incorporating FIB‐4, ELF, VCTE, and MRI‐based biomarkers may help identify patients most suitable for metabolic versus fibrosis‐targeted strategies, while enabling longitudinal monitoring without repeated liver biopsy [29, 31]. Importantly, several contemporary Phase 3 MASLD trials are already transitioning toward inclusion pathways and risk stratification based on noninvasive testing rather than mandatory biopsy alone, reflecting the broader movement toward scalable real‐world treatment models.

Ultimately, MASLD management is expected to evolve toward a personalized, multi‐drug, risk‐stratified approach tailored not only to fibrosis severity but also to cardiometabolic phenotype, obesity burden, diabetes status, and future liver‐related risk.

## Conclusion

MASLD has emerged as a complex, multisystem disease in which liver injury reflects the cumulative burden of metabolic dysfunction rather than an isolated hepatic disorder. Recent advances in noninvasive diagnostics, algorithm‐driven care pathways, and mechanism‐based therapeutics have fundamentally reshaped the clinical landscape, enabling earlier identification of at‐risk patients and more precise disease stratification. The convergence of metabolic unloading strategies, liver‐directed nuclear receptor modulation, and emerging anti‐fibrotic approaches now offers a realistic opportunity to alter disease trajectory, particularly when deployed within structured, sequential pathways and rational combination regimens. As the field moves beyond steatosis‐centric endpoints toward fibrosis regression and clinical outcomes, successful implementation will depend on integrating scalable diagnostics with therapies that address both systemic metabolic drivers and intrahepatic fibrogenesis. Together, these developments signal a transition from reactive management to proactive, precision‐based care, with the potential to substantially reduce the long‐term burden of MASLD at both individual and population levels. In the near term, the key implementation challenge is operational: selecting patients with the highest fibrosis risk, initiating therapies with complementary mechanisms, and monitoring durability, safety, and outcomes in real‐world pathways.

## Author contributions

*Concept and design of the study*: Philip N. Newsome and Saima Ajaz. *Literature research and clinical advice*: Philip N. Newsome and Saima Ajaz. *Critical revision of the manuscript for important intellectual content*: Philip N. Newsome.

## Conflict of interest statement

The authors declare no conflicts of interest.
